# The roles of cellular protease interactions in viral infections and programmed cell death: a lesson learned from the SARS-CoV-2 outbreak and COVID-19 pandemic

**DOI:** 10.1007/s43440-022-00394-9

**Published:** 2022-08-23

**Authors:** Martyna Majchrzak, Marcin Poręba

**Affiliations:** Department of Chemical Biology and Bioimaging, Faculty of Chemistry, Wroclaw University of Science and Technology, Wyb. Wyspianskiego 27, 50-370 Wroclaw, Poland

**Keywords:** SARS-CoV-2, COVID-19, Proteases, Cell death, Apoptosis, Pyroptosis, Lysosomes, Caspases, Cathepsins

## Abstract

The unprecedented pandemic of SARS-CoV-2 (severe acute respiratory syndrome coronavirus 2), which leads to COVID-19, is threatening global health. Over the last 2 years, we have witnessed rapid progress in research focusing on developing new antiviral vaccines and drugs, as well as in academic and clinical efforts to understand the biology and pathology of COVID-19. The roles of proteases among master regulators of SARS-CoV-2 invasion and replication and their pivotal roles in host defence against this pathogen, including programmed cell death, have not been well established. Our understanding of protease function in health and disease has increased considerably over the last two decades, with caspases, matrix metalloproteases, and transmembrane serine proteases representing the most prominent examples. Therefore, during the COVID-19 pandemic, these enzymes have been investigated as potential molecular targets for therapeutic interventions. Proteases that are responsible for SARS-CoV-2 cell entry and replication, such as TMPRSS2, ACE2 or cathepsins, are screened with inhibitor libraries to discover lead structures for further drug design that would prevent virus multiplication. On the other hand, proteases that orchestrate programmed cell death can also be harnessed to enhance the desired demise of infected cells through apoptosis or to attenuate highly inflammatory lytic cell death that leads to undesired cytokine storms, a major hallmark of severe COVID-19. Given the prominent role of proteases in SARS-CoV-2-induced cell death, we discuss the individual roles of these enzymes and their catalytic interactions in the pathology of COVID-19 in this article. We provide a rationale for targeting proteases participating in cell death as potential COVID-19 treatments and identify knowledge gaps that might be investigated to better understand the mechanism underlying SARS-CoV-2-induced cell death.

## Introduction

Coronaviruses are a large family of enveloped, positive-sense single stranded RNA viruses capable of infecting mammalian species, including humans [1]. Their genomes comprise open reading frames (ORFs) encoding non-structural and accessory proteins and four major structural proteins, envelope (E), membrane (M), nucleocapsid (N) and spike (S), which all have attracted considerable research attention due to their prominent roles in host cell invasion [2]. The recently emerged severe acute respiratory syndrome coronavirus 2 (SARS-CoV-2) has spread worldwide, causing coronavirus disease 2019 (COVID-19), which has become a serious global medical and economic threat [3]. The pathogenesis of COVID-19 is related to virus replication and infection, followed by inflammatory responses, which manifest as a cytokine storm in a severe course of illness that is characterized by elevated levels of IL1-β, IL-6, IL-8, ferritin and tumour-necrosis factor alpha (TNF-α) [4, 5]. Human coronaviruses, including highly pathogenic SARS-CoV-2, replicate in the lower respiratory tract and lead to acute respiratory distress syndrome (ARDS), a severe pneumonia-like syndrome. Two major hallmarks of severe COVID-19 are a persistent interferon response and sustained presence of viral RNA for months [6]. Therefore, multiple approaches have been proposed to combat COVID-19, including blocking the inflammasome response, downstream interferon response or viral replication [7–9]. Regardless of the strategy, triggering programmed cell death (PCD) either by the immune system itself or through therapeutic intervention is the centre of anti-COVID-19 action. PCD is a well-known innate host defence mechanism against intracellular pathogens that aids host defences in protecting against pathogenic microorganisms by destroying infected cells and thereby limiting intracellular pathogen survival and growth [10]. Based on accumulating evidence, diverse viral infections result in pyroptosis, apoptosis or necroptosis [11]. These processes are mainly orchestrated by caspases, which are cysteine proteases that are stored in the cytosol as inactive zymogens and undergo cascade activation upon exposure to various stimuli [12–14]. However, caspases are not the only proteases that are engaged in cell death. To date, multiple proteases have been identified to interact with these enzymes, either promoting or attenuating cell death [15–17]. This network is complex and strongly depends on external and internal stimuli, some of which are exclusive to viral infections. In this review, we provide insights into the roles of caspases and other proteolytic enzymes in the cell death process initiated by SARS-CoV-2 infection and discuss the pharmacological potential of modulating protease activity to combat COVID-19. Moreover, since the invasion mechanisms of multiple viruses, including coronaviruses, converge at common points, the substantial amount of research performed during the SARS-CoV-2 outbreak might be directly translated into the development of effective treatments for existing viral infections or viruses that are yet to come.

### The roles of host cellular proteases in SARS-CoV and SARS-CoV-2 invasion

**Fig. 1:**
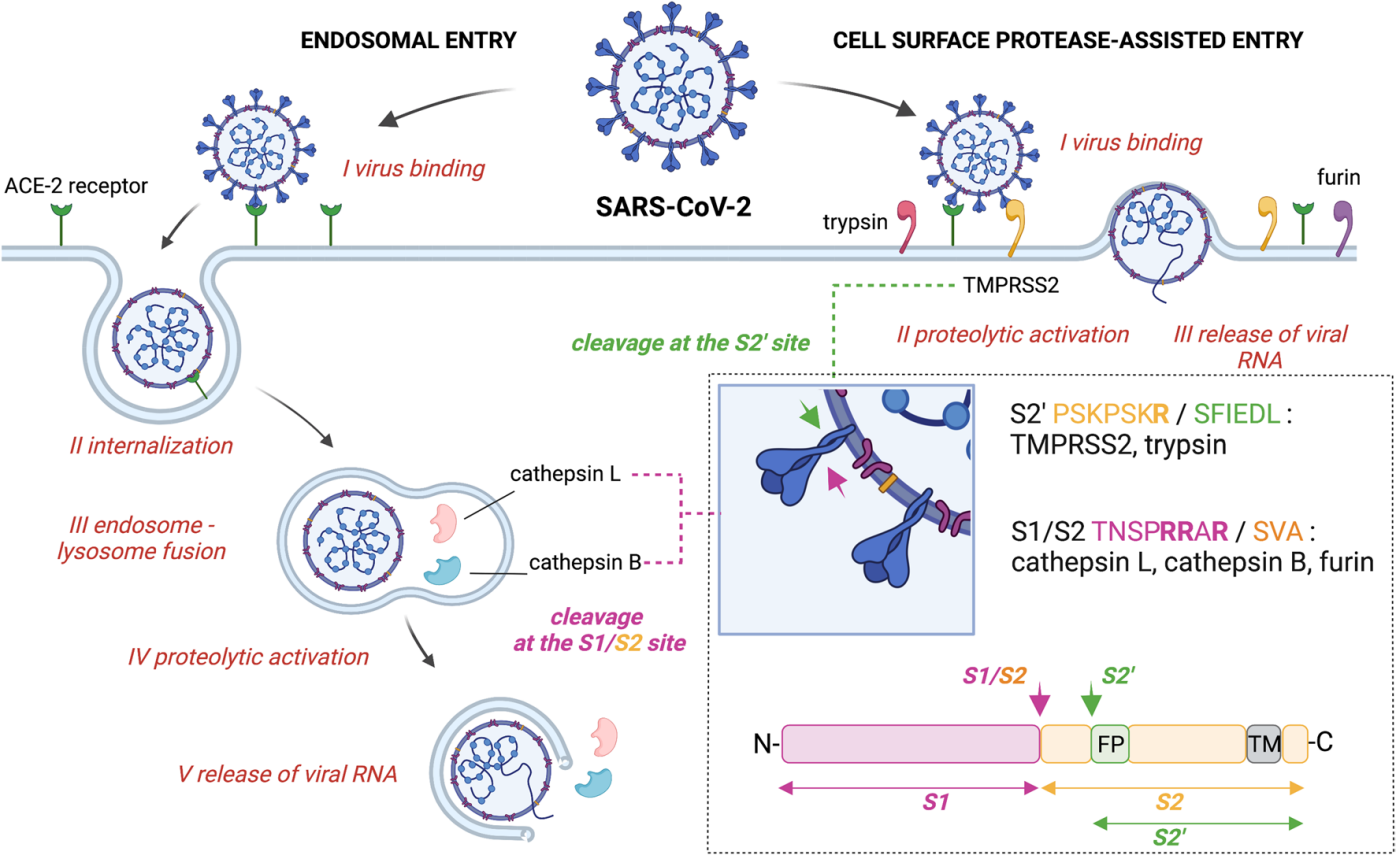
SARS-CoV-2 entry mechanisms. Endosomal pathway begins when viral coat spike protein binds to ACE2 receptor on the surface of host cell. Next, the virus is internalized through endocytosis into endosomes following by fusion with lysosomes, where spike protein is processed and activated by lysosomal proteases, cathepsin L and cathepsin B. Finally viral RNA is released and translated in the endoplasmic reticulum (ER) to form new SARS-CoV-2 virions. In the protease-assisted cell surface pathway SARS-CoV-2, upon binding to ACE2 receptor, is hydrolysed and activated by TMPRSS2 and other membrane serine proteases. This results in the direct release of viral RNA to the cytosol, relocation to ER and translation into new SARS-CoV-2 copies. The mechanism of proteolytic activation of spike protein by cysteine and serine proteases is presented in dashed box. Peptide sequences that are selectively recognized by cellular proteases are presented in single-letter amino acid code

Proteases constitute one of the largest groups of enzymes and are responsible for the catalytic hydrolysis of peptide bonds in peptide and protein substrates [18]. These enzymes are thus essential for virtually all biological processes; however, the deregulation of their activity is also associated with a multiple diseases, including impaired host defences against pathogens [19]. Viral and human proteolytic enzymes are now well established among the key regulators of both viral infections and the immune response [20, 21]. In a broad spectrum of viral infections, proteases remain the first line of host defences, including neutrophil serine proteases (human neutrophil elastase, cathepsin G, proteinase 3, neutrophil serine protease 4/NSP4, and granzymes) that contribute to the formation of neutrophil extracellular traps (NETs) or caspases in cytotoxic lymphocytes that orchestrate programmed cell death [22, 23]. This defence mechanism is further supported by cysteine proteases known as cathepsins and other proteases that contribute to antigen presentation and the immune response [24]. In contrast, human proteases are also used by the virus for invasion and replication. While the pathogenesis and tissue tropism of coronavirus are initially controlled by the interactions between spike (S) protein and host cell surface receptors, the direct proteolytic activation of spike by a broad arsenal of proteases plays a dominant role in subsequent host cell entry and invasion [25]. In early work on coronaviruses, researchers established that secreted-type serine and metallo-dependent proteases such as transmembrane serine protease 2 (TMPRSS2) and angiotensin-converting enzyme 2 (ACE2) process viral proteins, allowing the virus to enter the cell [26], and it is well established now that these enzymes are involved in the so called canonical pathway of SARS-CoV-2 invasion [26, 27]; however, some alternative noncanonical and nonproteolytic routes have also been identified [28–30] (Fig. 1). To date, multiple human proteases have been shown to be utilized by the virus in this Trojan horse strategy, and in most of these cases, the specific cleavage sites in viral proteins have already been identified [31]. Although several common cleavage patterns across various viruses have been revealed, some other interactions between host proteases and viral proteins are virus specific, underlying the need for further research. In 2002, the severe respiratory syndrome coronavirus (SARS-CoV), a member the family *Coronaviridae*, outbreak occurred and instantly gained considerable scientific attention [32]. SARS-CoV cell invasion was mediated by the engagement of ACE2 followed by either (a) endosomal internalization and subsequent processing by lysosomal proteases or by (b) extracellular limited proteolysis mediated by trypsin, thermolysin and other proteases [33]. Notably, protease-mediated invasion was 100- to 1000-fold more efficient than the endosomal pathway, highlighting the ultimate role of these enzymes in SARS-CoV infection and explaining why serine protease-rich tissues such as the respiratory tract are more prone to infection [33]. In subsequent research, several other proteases have also been identified to be important for virus–cell fusion by cleaving coronavirus spike protein (S) either within lysosomes and endosomes (cathepsin L and cathepsin B), granulocytes (elastase), cytosol and endosome (calpain-1) or extracellularly (furin, TMPRSS2, and human airway trypsin-like protease/HAT) [31, 34, 35]. Although the SARS-CoV outbreak collapsed in 2004, it reinforced research in coronavirus biology. A significant research effort that has been made since the new species of coronavirus SARS-CoV-2 appeared, confirmed that multiple proteases are directly engaged in cascade events from viral infection and cell entry to the host response and clearance of virus material through various cell death pathways, which mirrored the findings on SARS-CoV [26, 36]. However, since SARS-CoV-2 has become a considerably larger medical threat, research efforts are focused on antiviral therapies, some of which are designed to block the catalytic activity of proteolytic enzymes that are directly involved in viral invasion and replication [180].

### The role of cellular proteases in programmed cell death during SARS-CoV-2 infection

Regulated or programmed cell death is the final step of a plethora of biological processes, such as cell growth and differentiation and viral infections, but it is also a desired outcome of multiple therapies, including pharmacological, immune- or gene-based therapies [37, 38]. Understanding the molecular mechanisms of various cell death types is an absolute requirement for the design of safe and efficient treatments against diseases and allows us to better explain the biological functions of individual proteins. According to current research, several types of cell death have been identified, including apoptosis, necrosis, necroptosis, pyroptosis, NETosis, autophagy-dependent cell death, and lysosome-dependent cell death [37]. Some of them are accidental events triggered by nonselective factors such as high pressure, high temperature, or extreme pH, whereas others are tightly controlled by multiple proteins and small molecules. One of the major group of proteins that orchestrate programmed cell death are proteases. However, the proteolytic network in COVID-19 is complex, as proteases depend on the microenvironment and display pleiotropic activity. The large amount of COVID-19 research conducted over the last three years has provided new evidence of protease-specific roles in the infection and host response, as ultimately manifested by inflammation and programmed cell death [39]. These processes have been proposed to be major drivers of COVID-19 pathology. Moreover, severe COVID-19 is characterized by dysregulation of the cytokine release profile, which is either activated or attenuated by proteolytic events [40]. Therefore, researchers have postulated that pharmaceuticals targeting host cell programmed cell death pathways might be a very beneficial therapeutic strategy for mitigating the severe course of COVID-19 [41, 42]. Indeed, in individuals with COVID-19, apoptosis, necroptosis, and pyroptosis, which are all controlled by caspases, cooperate and coordinate innate and adaptive immune responses and inflammation, providing an attractive therapeutic avenue for pharmacological intervention [43–45]. On the other hand, serine neutrophil proteases and lysosomal proteases that control NETosis and lysosome-dependent cell death, respectively, are also among the master proteolytic regulators of SARS-CoV-2 invasion and the immune response [46–49]. Therefore, a better understanding of their interactions and cascade activation in response to COVID-19 may yield novel therapeutic avenues.

### NETosis

**Fig. 2:**
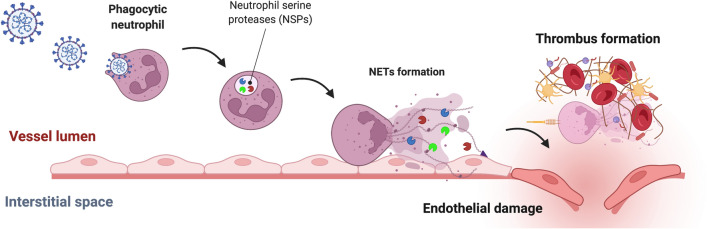
The schematic representation of NETs formation upon SARS-CoV-2 infection of neutrophils that ultimately lead to thrombosis in severe course of COVID-19 disease. Neutrophil serine proteases play major role the self-demise of neutrophils

Neutrophils are the most abundant leukocytes in the circulation and play major roles in the first line of innate immune responses to various infections [50]. They engulf and destroy microorganisms through intracellular degradation mediated by proteases, release of granules and induce the formation of neutrophil extracellular traps (NETs) [51]. According to the long-standing dogma, neutrophils constitute a homogenous population of terminally differentiated cells. However, a growing body of literature has shifted this view, and these cells are generally understood to present substantial phenotypic heterogenicity and functional versatility [52–54]. For instance, neutrophils express a very diverse distribution of proteases, including four neutrophil serine proteases (NSPs; neutrophil elastase, cathepsin G, proteinase 3, and NSP4), released by activated neutrophils, and various intracellular granzymes that coordinate cell death and survival, thus playing key roles in SARS-CoV-2 infection and the modulation of inflammation and the immune response [55, 56] (Fig. 2). At the onset of the COVID-19 pandemic, elastase and cathepsin G were highly upregulated in nasopharyngeal swabs from SARS-CoV-2-infected patients, which was directly associated with neutrophil degranulation and NETosis [57]. Indeed, severe COVID-19 is linked to excessive neutrophil function, as those patients have elevated levels of proteins associated with the formation of NETs and increased basal activation of NETs [58–60]. The upregulation of NETosis and, in some cases, neutrophil necroptosis is a consequence of the intensified infiltration and activation of neutrophils to the lungs, which interestingly, might be the effect of direct neutrophil infection or indirect mechanisms mediated by other immune cells through proinflammatory cytokine activation [60, 61]. Regardless of the activation mechanism, enhanced NETosis causes inflammation-directed coagulopathy, directly contributing to the high mortality of COVID-19 [58, 62, 63] (Fig. 2). Therefore, the inhibition of NET formation, for instance, through the inhibition of neutrophil elastase, might be a good therapeutic strategy to combat the severe course of COVID-19 [64, 65]. Another hallmark of SARS-CoV-2 infection that is at least partially orchestrated by neutrophils is the increased production of reactive oxygen species (ROS), which disrupts cellular homeostasis and ultimately leads to apoptosis or less controlled necrosis [66]. Apart from the cytokine-dependent immunomodulatory crosstalk between neutrophils and other cells resulting in NET formation or ROS generation, proteases also exert a direct effect on SARS-CoV-2 infection. Similar to monocytes and macrophages, ACE2- and TMPRSS2-positive neutrophils are more susceptible to SARS-CoV-2 entry, which was attenuated in vitro by the administration of ACE2-specific therapeutic antibodies or TMPRSS2 active site inhibitors [60, 67]. This observation indicates that ACE2 and TMPRSS2 are common receptor proteases for SARS-CoV-2 invasion in various cells. However, the role of neutrophil-specific proteases in COVID-19 has also been investigated. In a multicentre study of 155 patients, Gueant and co-workers observed that blood neutrophil elastase levels, along with histone-DNA and myeloperoxidase-DNA levels, was markedly increased in patients with COVID-19 compared to controls [68]. Moreover, the elevated elastase activity was positively correlated with increased levels of the IL-6, IL-8 and CXC motif chemokine receptor 2 (CXCR2) proteins, which fully reflected in vitro observations that neutrophils stimulated with IL-8 and CXCR2 displayed excessive NET formation and elastase activation [68]. Finally, neutrophil elastase was an independent marker of COVID-19-related lung damage in a computed tomography (CT) scan [68]. In healthy neutrophils, the activity of serine proteases, including neutrophil elastase, is tightly regulated by the presence of α-1 antitrypsin (A1AT), which is the major endogenous protease inhibitor [69]. Therefore, the variable expression of A1AT in individuals with different pathological states may compensate for the excess elastase activity. However, Zerimech and co-workers documented a disturbed balance between A1AT and elastase levels in patients with severe COVID-19, as the serum A1AT level was markedly reduced [70]. This change in turn leads to uncontrolled elastase activity, resulting in ARDS in those patients and suggesting the prominent role of elastase in COVID-19. Therefore, the pharmaceutical inhibition of elastase with already approved anti-elastase drugs (i.e., sivelestat or alvelestat) might be a valid option for the treatment of COVID-19, which has already been proposed in several studies [64, 71, 72]. Importantly, the inhibition of neutrophil elastase might also enhance the immune response to injected vaccines by supporting antibody production. Excess elastase not only enhances unwanted neutrophil cell death but also blunts the adjuvant activity of alum [65]. On the other hand, the in vitro study by Mustafa et al. showed that secreted elastase and proteinase 3, but not cathepsin G, directly cleaves the peptide bond adjacent to the polybasic amino acid side sequence at the SARS-CoV-2 spike protein S1/S2 activation loop [73]. This proteolytic event potentially leads to the priming of the S1/S2 interface during the immunological response, which is a key process for the virus to invade the cell [73]. The roles of proteinase 3 and cathepsin G in SARS-CoV-2 infection and related cell death are significantly less well studied, as most studies have focused on elastase; therefore, further efforts are needed to link the individual activities of these enzymes to COVID-19 pathology [74, 75]. The heterogenicity of neutrophils might play an essential role in COVID-19. Several studies have already reported that low-density neutrophils (LDNs) are markedly increased in patients with severe COVID-19 [76, 77]. However, further studies are needed to elucidate whether the distribution and activity of NSPs in LDNs differs from those in other neutrophil populations.

### Apoptosis

**Fig. 3:**
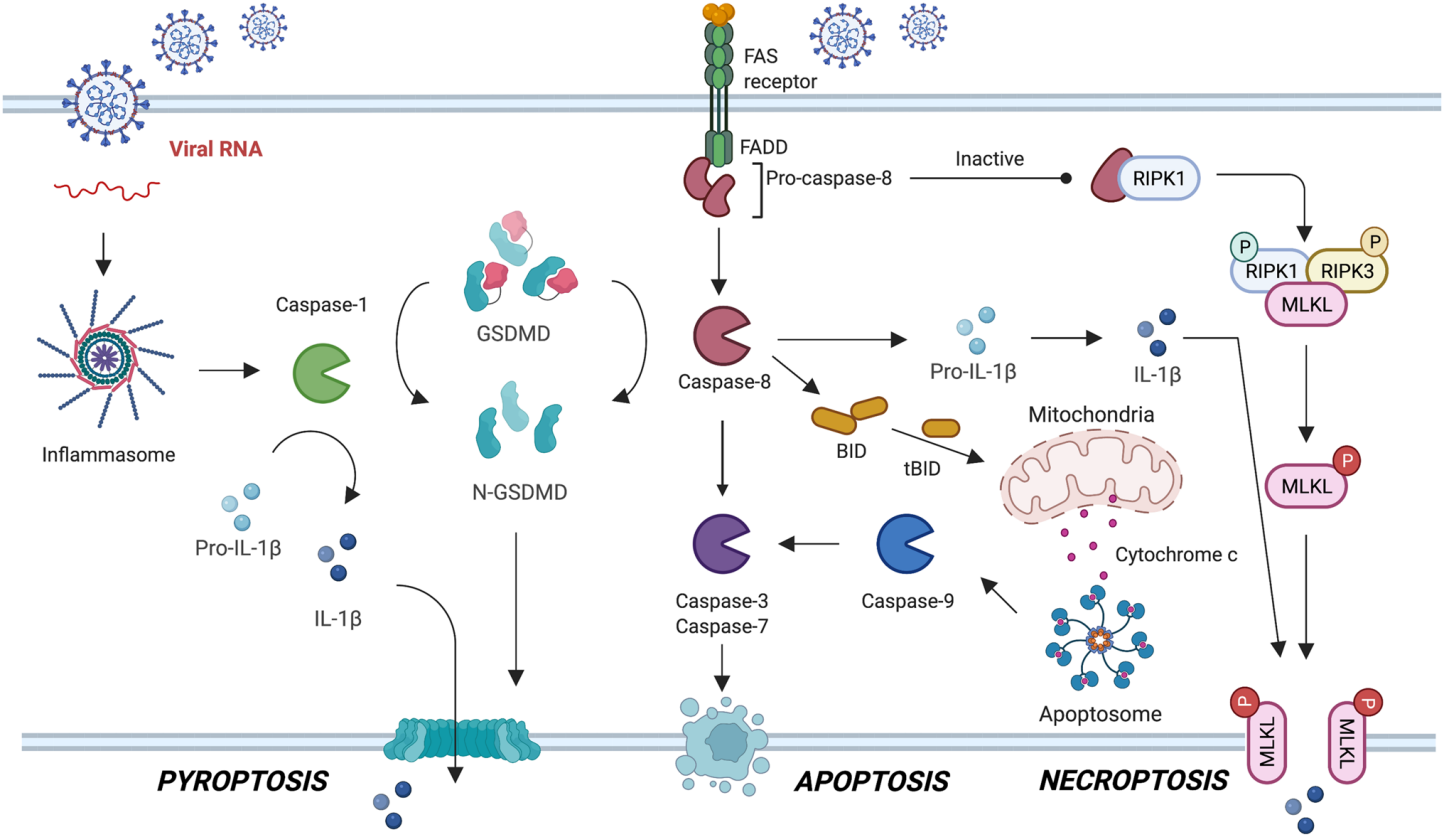
A simplified model of caspases activation upon SARS-CoV-2 cell infection. SARS-CoV-2 can induce cell death through multiple mechanisms, including pyroptosis, apoptosis and necroptosis. The activation of inflammasome through NF-κB pathway leads to the activation of caspase-1 that hydrolyses gasdermin D and proinflammatory cytokines, resulting in lytic cell death. Apoptosis is triggered extracellularly (DISC and caspase-8) or intracellularly (via apoptosome and caspase-9), leading to caspase-3 activation. The catalytic blockade of caspase-8 switches the cell death from apoptosis to pyroptosis through RIPK1/RIPK3 activation and MLKL phosphorylation. In parallel, active caspase-8 can cleave pro-interleukins enhancing inflammatory signals. The proteolytic crosstalk of caspases can also lead to the PANoptosis which occurs when multiple cell death pathways are triggered simultaneously

Apoptosis is the most well-studied type of regulated cell death, with caspases (-3, -6, -7, -8, -9, and -10) playing central roles in this cascade event [78–80]. Caspases are a highly conserved family of cytosolic cysteine proteases that display narrow substrate specificity by cleaving substrates after Asp residues [81, 82]. This proteolytic activity that is limited to single cleavage or a discrete pattern of a few cleavages in target substrates allows caspases to orchestrate apoptosis, one of the most ancient biological processes [83]. Apoptosis is a type of immunologically silent, nonlytic cell death that results in the formation of apoptotic bodies that are engulfed by macrophages. Therefore, infected, damaged or ageing cells are dismantled and safely removed from the organism [84]. Apoptosis can occur in all immune and nonimmune cells, highlighting the major role of this process in pathogen invasion, including SARS-CoV-2 infection and subsequent COVID-19 pathology. Apoptosis is mediated by external (caspase-8 and -10) or internal (caspase-9) pathways, and these processes converge at a common point, the activation of executioner caspases (caspase-3, -6, and -7) [85]. In the extrinsic apoptosis pathway, death ligands, mainly belonging to the superfamily of TNFs, bind to their cognate death receptor, leading to the recruitment and activation of the initiators caspase-8 and caspase-10 through their death effector (DED) domains within the death-inducing signalling complex (DISC) [86, 87]. On the other hand, intrinsic apoptosis is mainly triggered by the activation of B-cell lymphoma (Bcl-2) proteins that bind to the mitochondrion and cause mitochondrial outer membrane permeabilization (MOMP), resulting in the leakage of cytochrome C and subsequent activation of the initiator caspase-9 through its N-terminal caspase activation and recruitment domain (CARD) within the apoptosome that further activates downstream executioner caspases [86, 87]. Regardless of the apoptotic pathway, initiator caspases are activated through dimerization and proteolytic processing, which is called proximity-induced autoactivation [14]. Notably, the extrinsic and intrinsic pathways may be activated simultaneously to amplify the apoptotic signal. Moreover, the apoptotic network is also modulated by other proteases, including cytosolic calpains and lysosomal cathepsins [88, 89]. The role of apoptosis in viral infection has been well established, providing solid foundations for the study of SARS-CoV-2. In general, the apoptosis of infected cells is among the most efficient strategies to reduce the viral replication rate and ultimately eliminate the virus from the organism [90]. SARS-CoV-2 activates apoptosis via diverse mechanisms, which depend on the strain of the virus and mutations in its proteins [91–93]. Although the activation of caspase-3 is the major hallmark of all these processes, the upstream cascade events are more diverse [43, 44] (Fig. 3). The apoptotic pathways in SARS-CoV-2 infections must be carefully dissected, as commonly used small-molecule caspase inhibitors, such as LEHD-fmk, IETD-fmk, or DEVD-fmk, lack selectivity [94, 95]; therefore, the conclusions might be misleading, not only within the apoptotic cascade but also between apoptosis (caspases-3, -8, -9, -10) and pyroptosis (caspases-1, -4, -5) pathways, which might be blocked by the same inhibitors [93]. Although a growing body of literature reports the role of elevated apoptosis, as manifested by increased caspase-3 activity, in various immune and nonimmune cells of patients with COVID-19, only a few reports have elucidated the molecular mechanisms underlying this programmed cell death in SARS-CoV-2 infection [43, 96, 97]. Early in the COVID-19 outbreak, SARS-CoV-2 was shown to trigger the caspase-8-dependent death of lung epithelial cells [92]. For many years, caspase-8 was identified as a master regulator of apoptosis and necroptosis [98]; however, recent findings that this enzyme also participates in pyroptosis activation [99] clearly indicate that SARS-CoV-2-induced cell death is a complex process. Caspase-8 simultaneously triggers apoptosis but also directly activates pro-IL-1beta, which in turn triggers the necroptotic/pyroptotic pathway, resulting in an inflammatory response and the infiltration of immune cells [100]. The pleiotropic activity of caspase-8 in SARS-CoV-2 infection has been further investigated. Ren and co-workers showed that ORF3a, one of the accessory proteins expressed by SARS-CoV-2, induces apoptosis through caspase-8 activation [101]. In turn, activated caspase-8 processes pro-caspase-3 and activates pro-caspase-9 through Bid cleavage, which amplifies the apoptotic signal. Other SARS-CoV-2 ORF proteins have also been documented to induce cytotoxicity through apoptotic pathways, but their caspase-dependent mechanisms have not been investigated [102, 103]. A similar apoptotic amplification mechanism was described by Ren and co-workers [104]. The authors found that the SARS-CoV-2 membrane (M) protein triggers the activation of caspase-8 and caspase-9 through the downregulation of the phosphoinositide-dependent kinase-1 and protein kinase B (PDK1-PKB/Akt) signalling pathway. Importantly, although nucleocapsid (N), the other SARS-CoV-2 key structural protein, does not activate apoptosis by itself, it strongly enhances M-induced apoptosis, providing deeper insight into the pathogenicity of this virus. In another study, caspase-8 was upregulated in SARS-CoV-2-infected lung epithelial cells, resulting in enhanced pyroptosis [92]. Interestingly, the pharmaceutical inhibition of caspase-8 attenuated lytic cell death but did not alter viral replication. Although multiple studies have addressed the role of caspase 8 in SARS-CoV-2-induced apoptosis, the contribution of caspase-10 to this process is still enigmatic and has not been investigated.

### Pyroptosis

In contrast to apoptosis, pyroptosis is a lytic and highly inflammatory cell death pathway that is often called gasdermin D (GSDMD)-mediated programmed necrosis [105–107]. This type of cell death results in inflammation, as the cell membrane is disrupted and the cellular contents leak into the extracellular environment. Originally, pyroptosis was considered a protective mechanism, during which SARS-CoV-2-infected cells would be removed from the organism to prevent a productive viral cycle and enhance the immune response to the virus [108, 109]. However, in some cases, progressive pyroptosis leads to hyperactivation of the immune system, leading to a cytokine storm and resulting in a severe course of COVID-19 manifested by lung injury and multiple organ failure [42, 110–113]. Pyroptosis is governed by caspases (-1, -4, and -5 in humans and -1 and -11 in mice) that convert pro-ineteleukins-1β and -18 into active cytokines and activate GSDMD [114, 115]. Canonical pyroptosis is orchestrated by caspase-1 [105], whereas noncanonical pyroptosis is triggered by caspase-4 in humans and caspase-11 in mice [107]. Regardless of the type of pyroptosis, inflammatory caspases-1, -4, -5, -11 cleave gasdermin D at the FLTD275 site (LLSD276 for mouse gasdermin D) to disrupt autoinhibitory interactions, thus allowing the GSDMD N-terminal domain to oligomerize on the cell membrane and form pores, resulting in the release of cytosolic contents outside the cell [105–107]. Recently, three independent studies reported that caspase-8, which was previously recognized as an apoptotic enzyme, directly cleaves GSDMD at the same site as caspase-1, thereby stimulating pyroptosis even in the absence of classical proinflammatory caspases [116–118]. Therefore, caspase-8 is now recognized as a master regulator between apoptosis and pyroptosis. Indeed, in 2020, Zheng and co-workers documented that impaired NLRP3 inflammasome activation in murine coronavirus mouse hepatitis virus (MHV)-infected cells leads caspase-8 activation, followed by GSDMD or GSDME cleavage and robust lytic cell death [119]. In contrast, caspase-3 also cleaves gasdermin D, but the cleavage site at DAMD87 is different from the capase-1 processing site, which results in the generation of an inactive N-terminal fragment, attenuation of pyroptosis and increased apoptosis [120]. On the other hand, caspase-3 triggers pyroptosis by directly cleaving gasdermin E (GSDME), which has been observed in several cancer cell models with high GSDME expression [121–124]. This complex kinetic race, which depends on caspase and gasdermin expression and specific activation pathways, has considerable consequences for the fate of the cell [125]. Therefore, interest in harnessing GSDMD and GSDME as potential molecular targets for antiviral therapy has increased. Regardless of the type of virus, the cellular pathways converge at the common point at which caspases cleave and activate gasdermins. According to the classical view, SARS-CoV-2 infection results in the activation of the Nod-like receptor family pyrin domain-containing 3 (NLRP3) inflammasome pathway, resulting in the sequential activation of caspase 1 and the inflammatory cytokines pro-IL-1beta and pro-IL-18 and GSDMD [42] (Fig. 3). This process results in pyroptotic cell death manifested by the leakage of cell contents along with IL-1beta, IL-18 and damage-associated molecular patterns (DAMPs) to the extracellular milieu [42, 110–113, 126]. Moreover, the active form of caspase-1 (p20 subunits) was detected in peripheral blood mononuclear cells (PBMCs) and sera from patients with COVID-19, directly indicating the mechanism of pro-interleukin activation [126]. The release of active IL-1beta induces the influx and activation of neutrophils, as well as the activation of T- and B-cells and the differentiation of Th17 cells, whereas IL-18 induces a downstream interferon gamma (IFN-γ) response. Therefore, these interleukins are among the major proteins involved in innate immunity and adaptive immune responses. As shown in a recent study, activation of the inflammasome in SARS-CoV-2-infected lung-resistant macrophages is the major driver of COVID-19, and the subsequent inhibition of the inflammasome with small molecules targeting NLRP3 or caspase-1 reverses chronic lung pathology [127]. Since the release of interleukins is strongly depends on GSDMD, the elevated expression of this protein is associated with an increased risk of severe COVID-19, indicating a critical role for this pathway in the pathogenesis of SARS-CoV-2 infection. On the other hand, caspase-3-dependent GSDME hydrolysis, which also triggers pyroptosis, might also contribute to the severity of COVID-19 [119]. Recent reports by Ma indicate that multinucleated syncytia formed by the fusion of SARS-CoV-2 spike and ACE2-expressing cells undergo lytic cell death in patients with severe COVID-19 through the activation of the caspase-9/caspase-3 pathway, ultimately leading to pyroptosis [128]. This proteolytic crosstalk between apoptosis, pyroptosis and, to some extent, necroptosis is a hallmark of PANoptosis, which is defined as an inflammatory programmed cell death regulated by PANoptosome, a complex containing key features of individual cell death pathways, including the NLRP3 complex (pyroptosis), caspase 8 machinery (apoptosis) and receptor-interacting serine/threonine-protein kinase 1 (RIPK1) and 3 (RIPK3) regulators (necroptosis) [129]. The importance of PANoptosis in COVID-19 pathology was observed by treating mouse bone marrow-derived macrophages with both TNF-alpha and IFN-gamma to activate the Janus kinase/signal transducer and activator of transcription 1/interferon regulatory factor 1 (JAK/STAT1/IRF1) pathway to drive caspase-8/FADD-mediated PANoptosis [130]. The combined application of these cell death stimuli mirrored the COVID-19 cytokine storm, and the application of TNF-alpha- and IFN-gamma-neutralizing antibodies protected against SARS-CoV-2 in mice. Although the roles of the NLPR3 inflammasome in the induction of pyroptosis and PANoptosis in COVID-10 pathology is well established, researchers have postulated that other Nod-like receptors (NLRs) might contribute to this process. This hypothesis is partially supported by the fact that capase-1 inhibition resulted in a stronger anti-pyroptotic effect than the inhibition of NLPR3 alone, suggesting other upstream pathways of caspase-1 activation. Indeed, a recent study reported that SARS-CoV-2 infects blood monocytes and leads to the parallel activation of NLRP3, Nod-like CARD domain-containing protein 4 (NLRC4) and absent in melanoma protein 2 (AIM2) inflammasomes, indicating that this virus engages multiple inflammasome sensors simultaneously [131]. Finally, granzymes might also orchestrate SARS-CoV-2-induced pyroptosis of cytotoxic T lymphocytes and natural killer cells (NK cells), as these enzymes are known to directly cleave and activate gasdermin B (granzyme A) and gasdermin E (granzyme B); both events result in pore formation [122, 132].

### Necroptosis

The major hallmark of apoptosis and pyroptosis is the hierarchical activation of caspases that perform limited proteolysis of their endogenous substrates to induce cell death. However, when the cell receives a death signal but caspase activity is silenced, the cell switches to necroptosis, which is defined as necrotic cell death dependent on RIPK3 [133]. Necroptosis was first observed when cells were treated with tumour necrosis factor in the presence of small-molecule inhibitors that target caspase-8 activity [134]. Although this process does not require caspase activity, it is still caspase-dependent, as inhibition of caspase-8 is important in the assembly of the ripoptosome, a death-inducing complex containing RIPK1, FADD and caspase-8 [135]. Moreover, necroptosis is blocked by small molecules such as necrostatin-1 (Nec-1), a direct RIPK inhibitor, indicating that this cell death pathway is well regulated and considered a form of PCD [136]. Although apoptosis and necroptosis share several upstream signalling factors and the execution of either signalling pathway is modulated by cellular inhibitors of apoptosis (IAPs) [137] or FLICE-like inhibitory protein (FLIP) [138], the ultimate outcome differs significantly, as necroptosis is a highly inflammatory process. Therefore, necroptosis is among the major host defence mechanisms activated to combat pathogens. In viral infections, including SARS-CoV-2 infections, necroptosis is triggered to release damage-associated molecular patterns (DAMPs) that recruit cytokine- and chemokine-producing immune cells [139] (Fig. 3). The prominent role of this process in SARS-CoV-2 infection has been confirmed in vitro, where the phosphorylation of mixed lineage kinase domain like pseudokinase (MLKL), a key necroptosis protein, was studied in Calu-3 human lung cancer cells, and clinical investigations showed that patients with severe COVID-19 display upregulation of RIPK3 proteins that trigger necroptosis signalling [140]. Nevertheless, based on accumulating evidence, necroptosis never occurs alone following SARS-CoV-2 infection, as it is always accompanied by apoptosis and/or pyroptosis.

### Lysosome-dependent cell death

**Fig. 4:**
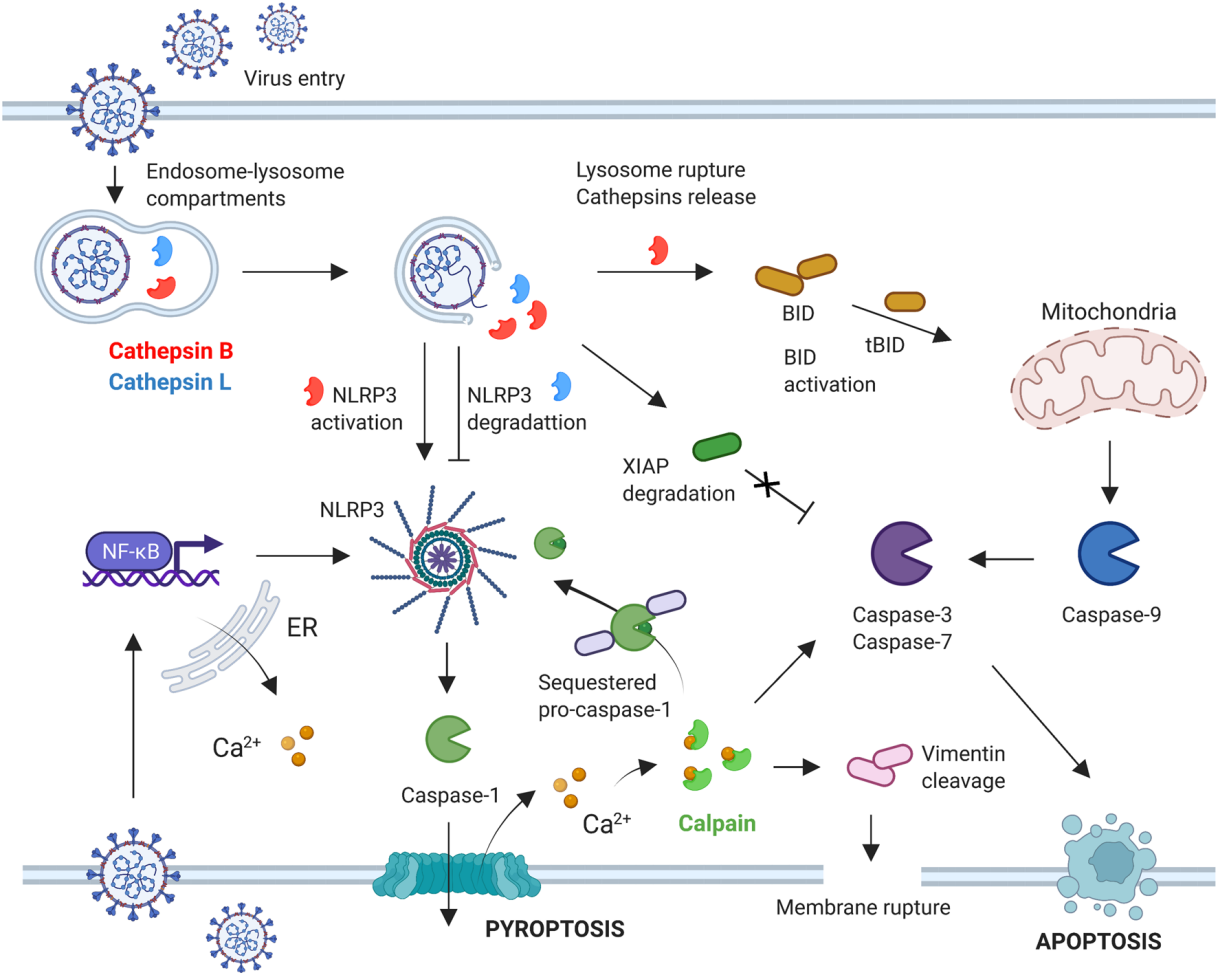
The putative proteolytic cross-talk between cysteine cathepsins, calpains (calpain-1 and calpain-2) and caspases in SARS-CoV-2 infected cells. Upon virus entry into the endosomal-lysosomal compartment, the lysosomes are disrupted resulting in cathepsins leakage into the cytosol. Next, cathepsin B might support pyroptosis by activating NLRP3 inflammasome, or enhance apoptosis by cleaving Bid protein, leading to caspase-9 activation, and degrading inhibitors of apoptosis proteins (IAPs). However, the excessive activity of lysosomal cathepsins (especially cathepsin L) may lead to the degradation of inflammasome components, preventing from NLRP3 assembly and activation. The virus entry also leads to increased concentration of calcium ions resulting in the activation of calpain-1. This protease enhances pyroptosis by releasing sequestered pro-caspase-1 from the cytoskeleton or support apoptosis by the activation of pro-caspase-3. Calpain-1 can also directly cleave vimentin, a cytoskeletal protein, leading to impaired mechanical resilience and membrane rupture

Caspases play a central role in programmed cell death; however, over the years, a number of other proteases, including cathepsins, calpains and granzymes, have also been reported to be involved in cell death. Cathepsins orchestrate the so-called lysosome-dependent cell death pathway [141]. In this mechanism, certain stimuli cause lysosome membrane permeabilization (LMP), resulting in the translocation of cysteine cathepsins into the cytosol [142]. Their broad substrate specificity enables cathepsins to perform stepwise degradation of target substrates; however, processing via limited proteolysis is also possible [143]. Cysteine cathepsins are mainly active in the acidic environment of the lysosomal-endosomal system, where they are primarily responsible for protein recycling and participate in antigen processing and presentation on major histocompatibility complexes (MHC) class II [144]. The disruption of large numbers of lysosomes leads to uncontrolled necrosis; therefore, cathepsins participate in programmed cell death only in the presence of moderate lysosomal lysis [145, 146]. According to some reports, cathepsins that have translocated to the cytosol are active for a certain period to cleave a panel of protein substrates, allowing them to participate in signal transduction. This process was confirmed for their role in apoptosis, as cathepsins, mainly cathepsin B, serve as apoptosis promotors by cleaving Bid and X-linked inhibitor of apoptosis protein (XIAP) [89, 147]. This process has been investigated in greater detail over the years. Under some pathological conditions, cathepsins clearly play an important role in apoptosis induction by destabilizing mitochondria [148–150]. The subsequent hypothesis that lysosomal cathepsins are also apoptosis executioners was rejected, as no downstream apoptotic proteins were identified to be cleaved by these enzymes. Since the role of cathepsins in apoptosis is well described, only limited data describing their contribution to the inflammatory process are available. In one such report, the authors showed that cathepsin B released into the cytosol helps activate caspase-1 through an interaction with NLRP3 [151]. Several subsequent studies confirmed the major role of cathepsin B in inflammasome assembly upon lysosome leakage [152–154]. Interestingly, cathepsin B is ubiquitously expressed, including in monocytes, alveolar epithelial cells and endothelial cells of the lungs and kidneys, which are primary targets for SARS-CoV-2 invasion [155–157]. Therefore, in SARS-CoV-2-infected cells undergoing pyroptosis, especially in monocytes and macrophages with high cathepsin B expression, this protease should participate in inflammasome activation through NLRP3 assembly (Fig. 4). The roles of other lysosomal cathepsins in pyroptosis are less studied, but several cathepsins still compensate for the loss of cathepsin B and trigger caspase-1-dependent inflammation [154]. Therefore, investigations of the function of cysteine cathepsins in COVID-19 not only in the context of their role in the processing of spike protein to facilitate the entry of viral nucleic acid into the human host cell but also in the context of cathepsin-mediated cell death are urgently needed. Multiple studies that focus on testing the anti-SARS-CoV-2 activity of cathepsin inhibitors have not explored cell death mechanisms; therefore, their role in modulating cell death might be overlooked [158–162].

## Calpain-1, the mediator between apoptosis, pyroptosis and lysosome-dependent cell death

The interactions between caspases and lysosomal cathepsins in cell death are complex. However, this system becomes even more complicated, as calpains (namely calpain-1 and calpain-2) may release cathepsins into the cytosol under some conditions, followed by caspase activation and cell death execution [163, 164]. Similar to cathepsins, calpains have also been shown to participate in apoptosis by serving as signal modulators of the caspase activation cascade. Calpain-1 is activated by micromolar concentration of calcium ions, therefore is much more abundant than calpain-2, which requires millimolar concentration of calcium ions for its activity. These Ca^2+^-activated neutral cysteine proteases were scientifically recognized earlier than caspases, but their roles in cell death are enigmatic [165]. These enzymes display broad substrate specificity; however, rather than nonselective digestion, they perform limited proteolysis, which may be crucial in pyroptosis and lysosome-dependent cell death [166]. Although the role of calpains in pyroptosis is not fully understood, recent reports indicate that this role is larger than previously appreciated. For instance, in pyroptotic cells, activated calpain-1 cleaves the structural protein vimentin, leading to the loss of intermediate filaments and the loss of mechanical resilience to subsequently facilitate cell rupture [167]. Calpain-1 also exhibits cross-talk with caspase-1, providing an amplification loop for inflammasome activation [168] (Fig. 4). NLRP3 is activated upon exposure to various insults, such as K^+^ efflux, release of mitochondrial ROS, or cytosolic Ca^2+^ influx, the latter of which is a prerequisite for calpain activation [115, 169]. Moreover, activated calpain-1 releases caspase-1 from its sequestration in the cytoskeleton, thereby increasing the caspase-1 concentration in the cytosol and enhancing inflammasome assembly and activity [168]. In SARS-CoV and SARS-CoV-2 infections, cytosolic Ca^2+^ influx and subsequent calpain activation are induced by the viral E protein and ORF3a [170, 171]. Therefore, in SARS-CoV-2-induced pyroptosis, calpain-1 is activated in parallel to capase-1 and contributes to this programmed cell death pathway. Reports on the role of calpains in COVID-19 are largely limited to the repurposing of their inhibitors, as some of these molecules were found to block the activity of the 3CL^pro^ SARS-CoV-2 protease [172–174]. Nevertheless, calpain-1 expression was downregulated in autopsy specimens from patients with type 2 diabetes infected with SARS-CoV-2 who developed acute cardiovascular syndrome [175]. This downregulation resulted in limited hydrolysis of junctophilin-2 (JP2), which is essential for maintaining junctional cardiac dyads and excitation–contraction coupling, and weakened the self-protective mechanism [176].

### Protease inhibitors modulating cell death to combat SARS-CoV-2 infection

To date, multiple studies have shown that proteases are among the key proteins involved in SARS-CoV-2 invasion and replication and play important roles in the development of COVID-19, influencing the course of this disease from mild infection to life-threating illness. In fact, proteases may either serve as pro- or anti-SARS-CoV-2 modulators, and their cell- and context-specific roles must be thoroughly investigated to develop anti-COVID-19 therapeutics. The most apparent strategy to combat COVID-19 through pharmaceutical intervention is to block the activity of proteases expressed by SARS-CoV-2 [177–179]. Recently, the first orally available drug, nirmatrelvir, which is an inhibitor of SARS-CoV-2 3CL^pro^ protease, was approved for the treatment of mild-to-moderate COVID-19 [180, 181]. Efforts to develop a drug for the second major SARS-CoV-2 protease, PL^pro^, are ongoing. [179, 182, 183]. However, during the COVID-19 outbreak, multiple other molecules that modulate the activity of cell death proteases have been extensively investigated. One of the most viable examples is inhibitors of inflammatory caspases, which are currently being repurposed as treatments for COVID-19. Since caspase-1 is a master regulator of inflammasome activation and subsequent IL-1beta activity, it appears to be a potential molecular target. Indeed, a classical pan-caspase inhibitor, emricasan, has been recently reported to reduce caspase-1 activity in CD4^+^ T cells from patients with COVID-19 ex vivo [43]. Other caspase-1 inhibitors, such as belnacasan, have also been evaluated for the treatment of patients with mild to moderate COVID-19 (clinical trials NCT05164120). Moreover, these molecules were also identified as potential inhibitors of 3CL^pro^ protease in an in silico screen, enhancing their potential clinical applications [184]. However, the considerable issue is the fact that although all these inhibitors were developed in the past decade and thoughtfully studied, none of them has ever received market approval due to hepatotoxicity. Therefore, the current efforts in anti-COVID-19 drug design targeting inflammasomes have shifted towards inhibitors of NLRP3 (DFV890, or dapansutrile, clinical trials NCT04382053) or gasdermin D (disulfiram [185], or dimethyl fumarate [186]), the latter of which is activated downstream of all inflammasome sensors. In the conventional view of SARS-CoV-2 infection, lysosomal cathepsins, mainly cathepsins B and L, are responsible for the activation of the spike protein by performing limited proteolysis at the S1/S2 and S2’ sites. Multiple studies have revealed that the pharmacological inhibition of cathepsins attenuates but does not fully inhibit viral infection and replication [160]. Therefore, the dual inhibition of cathepsins (endosomal–lysosomal virus entry pathway) and TMPRSS2 (extracellular pathway) exerts synergistic effects, which is of particular clinical importance because SARS-CoV-2 displays broad tissue tropism, requiring combining pharmaceutical strategies [187]. Several drugs targeting serine proteases that also inhibit TMPRSS2 protease have received market approval, i.e., camostat [188] or nafamostat [189], and these dual inhibitors appear to be a promising strategy. Unfortunately, to date, no single cathepsin inhibitor has been clinically approved for the treatment of cathepsin-related diseases, such as cancer or inflammatory disorders [190], which limits the initial enthusiasm for developing cathepsin-targeted COVID-19 medicines.

### Summary

Accumulating evidence has now indicated that proteases play major roles in SARS-CoV-2 infection and replication and are among the master regulators of COVID-19 severity. With the recent unprecedented success of manufacturing the first anti-COVID-19 drug, a 3CL^pro^ protease inhibitor, these enzymes have attracted considerable scientific attention. The majority of research efforts towards the development of new antiviral therapeutics are devoted to inhibiting host proteases that, by performing limited proteolysis, facilitate SARS-CoV-2 entry and replication. However, once COVID-19 progresses, attenuating the infection by modulating cell death is clinically beneficial. As programmed cell death is a desired outcome of various infections, under some circumstances, this type of cell death, especially pyroptosis, may lead to a cytokine storm, which is associated with a poor prognosis and high mortality rate. Since SARS-CoV-2 infection may activate various cell death pathways, including robust NETosis or PANoptosis, an understanding of the mechanisms underlying these processes is of paramount importance. The elucidation of the roles of individual proteases and their interactions might be key to proposing new anti-COVID-19 treatment options. Moreover, the therapeutic effect of modulating protease activity might be enhanced by targeting other sensors involved in their pathways; for example, the parallel inhibition of NLRP3 assembly along with gasdermin D oligomerization increases the therapeutic effect of caspase-1 inhibition to prevent pyroptosis. Since multiple proteolytic networks in cell death converge at common points, a comprehensive understanding of this machinery is necessary to better manage COVID-19.

## Data Availability

Not applicable.

## Abbreviations

A1AT: α-1 Antitrypsin
ACE2: Angiotensin-converting enzyme 2
AIM2: Absent in melanoma 2 protein
ARDS: Acute respiratory distress syndrome
Bcl-2: B-cell lymphoma protein
CARD: Caspase activation and recruitment domain
CXCR2: CXC motif chemokine receptor 2
COVID-19: Coronavirus disease 2019
CT: Computed tomography
DAMPs: Damage-associated molecular patterns
DED: Death effector domain
DISC: Death-inducing signalling complex
ER: Endoplasmic reticulum
FLIP: FLICE-like inhibitory protein
FMK: Fluoromethyl ketone
GSDMD: Gasdermin D
GSDME: Gasdermin E
HAT: Human airway trypsin-like protease
IAPs: Inhibitors of apoptosis
IFN-γ: Interferon gamma
IL: Interleukin
IRF1: Interferon regulatory factor 1
JAK: Janus kinase
JP2: Junctophilin-2
LDNs: Low-density neutrophils
LMP: Lysosome membrane permeabilization
MHC: Major histocompatibility complexes
MLKL: Mixed lineage kinase domain like pseudokinase
MOMP: Mitochondrial outer membrane permeabilization
MHV: Mouse hepatitis virus
Nec-1: Necrostatin 1
NETs: Neutrophil extracellular traps
NK cells: Natural killer cells
NLRs: Nod-like receptors
NLRC4: Nod-like CARD domain-containing protein 4
NLRP3: Nod-like receptor family pyrin domain-containing 3
NSP4: Neutrophil serine protease 4
NSPs: Neutrophil serine proteases
ORF: Open reading frames
PANoptosis: Pyroptosis, apoptosis, necroptosis programmed cell death
PBMCs: Peripheral blood mononuclear cells
PCD: Programmed cell death
PDK1: Phosphoinositide-dependent kinase-1
PKB/Akt: Protein kinase B
RIPK1: Receptor-interacting serine/threonine-protein kinase 1
RIPK3: Receptor-interacting serine/threonine-protein kinase 3
ROS: Reactive oxygen species
SARS-CoV-2: Severe acute respiratory syndrome coronavirus 2
STAT1: Signal transducer and activator of transcription 1
XIAP: X-linked inhibitor of apoptosis protein
TNF-α: Tumour necrosis factor alpha
TMPRSS2: Transmembrane serine protease 2
